# Supplementary material for: Metabolic control of smooth muscle cell phenotype switching in atherosclerosis
Source: Sci Adv. 2026 Aug 26;12(35):eaef6065. doi: 10.1126/sciadv.aef6065 (PMC13510649; doi:10.1126/sciadv.aef6065)
Supplement: Supplementary file 1 [file sciadv.aef6065_sm.pdf]

Science Advances
AAAS

Supplementary Materials for

Metabolic control of smooth muscle cell phenotype switching in atherosclerosis

Rong-Mo Zhang *et al.*

Corresponding author: Michael Simons, michael.simons@yale.edu

Sci. Adv. **12**, eaef6065 (2026)
DOI: 10.1126/sciadv.aef6065

This PDF file includes:

Figs. S1 to S5
Tables S1 and S2

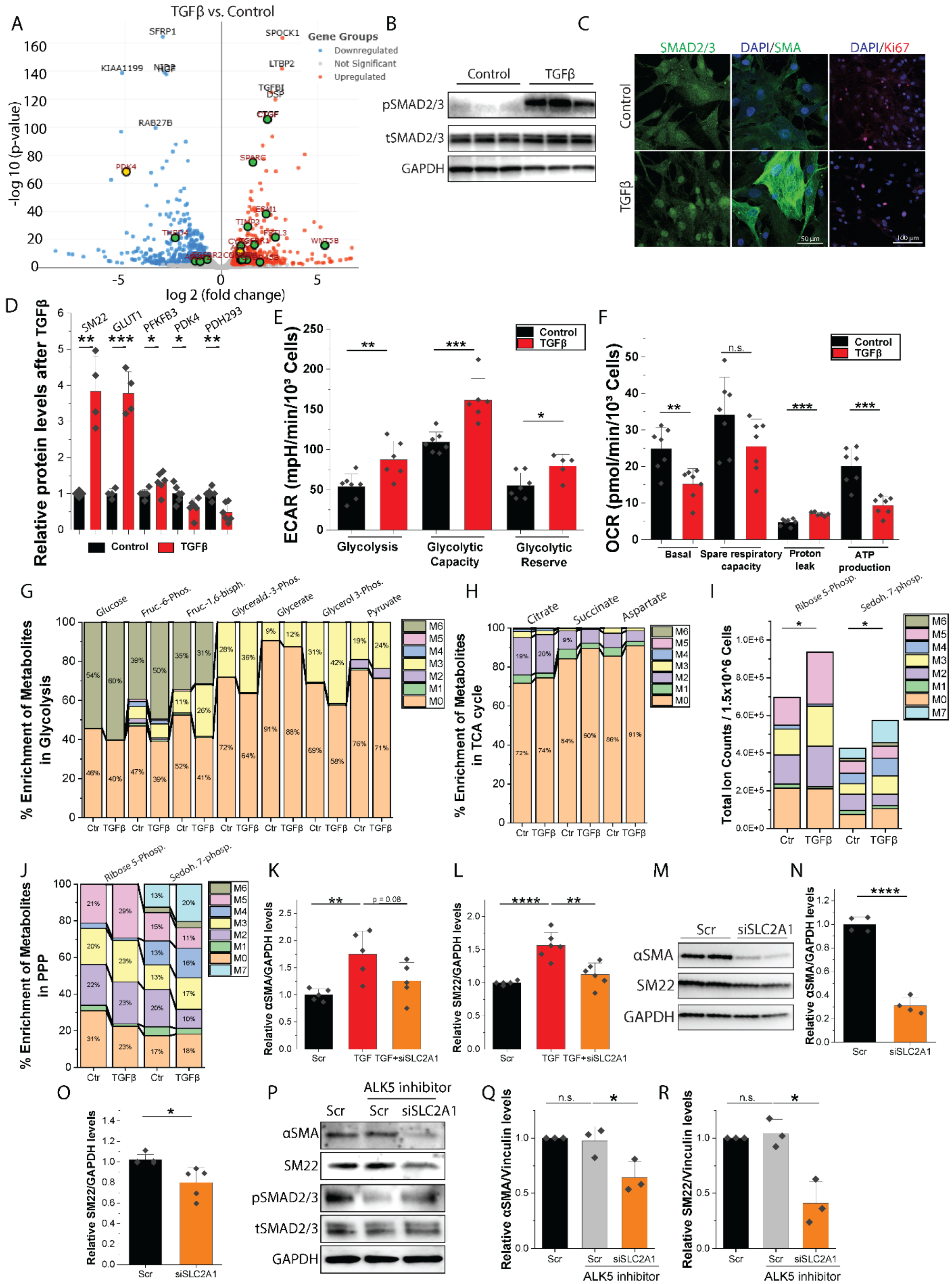

Supplementary figure 1 Metabolic effects of HASMC TGFβ signaling

(**A**) Volcano plot of transcriptome in TGFβ treated HASMC compared to control. Green dots represent TGFβ-targeted genes. Yellow dots represent glycolytic enzymes. Note that PDK4 is among the most downregulated genes after 7-day of TGFβ treatment (n = 3 technical replicates).

(**B**) Western blotting of TGFβ signaling (SMAD2/3 phosphorylation).

(**C**) Immunostaining of SMAD2/3, αSMA and Ki67. Note that TGFβ promotes the SMAD2/3 translocation to the nucleus, and induces HASMCs to a more contractile phenotype with increased αSMA and decreased Ki67.

(**D**) Quantification of SM22, GLUT1, PFKFB3, PDK4 and PDH293 after 7-day of TGFβ treatment. Normalized and pooled results from 4-5 independent repeats. Each dot represents one technical replicate.

(**E-F**) Seahorse analyses of (E) glycolysis, glycolytic capacity and glycolytic reserve and (F) basal oxygen consumption, spare respiratory capacity, proton leak and ATP production of HASMCs after treated with 10ng/mL TGFβ for 7 days. Representative results were displayed with 8 technical replicates. Each dot represents one technical replicate in one repeat.

(**G-J**) LC-MS metabolomics analysis of key metabolites in HASMCs treated with C^{13}-glucose (U-13C6-glucose, 10 mM) for 24 h after 7 days of TGF-β stimulation. M# represents the number of C^{13} -labeled carbon in each metabolite. Note that the % of C^{13} labelled metabolites in glycolysis (G) and pentose phosphate pathway (PPP, H-I) were increased by TGFβ, whereas that of key metabolites in the TCA cycle(H) were not increased. Representative results were displayed with quadruplicates each time.

(**K-L**) Quantification of αSMA and SM22 levels, normalized to GAPDH, to investigate the effects of GLUT1 KD (siSLC2A1) on HASMC contractile phenotype under 10 ng/mL TGFβ treatments for 3 days. Normalized and pooled results from 3 independent repeats. Each dot represents one technical replicate.

(**M-R**) Representative western blots and quantification studying the effects of GLUT1 KD (siSLC2A1) on HASMC contractile phenotype (αSMA and SM22) under normal growth medium, with baseline TGFβ level (M-O), or with ALK5 inhibitor treatment for 3 days (P-R). Normalized and pooled results from 3 independent repeats. Each dot represents one technical replicate.

One-way ANOVA was used for statistical analyses in (D, E, F, I, K, L, N and O). Paired-sample t-test was used in (Q and R).

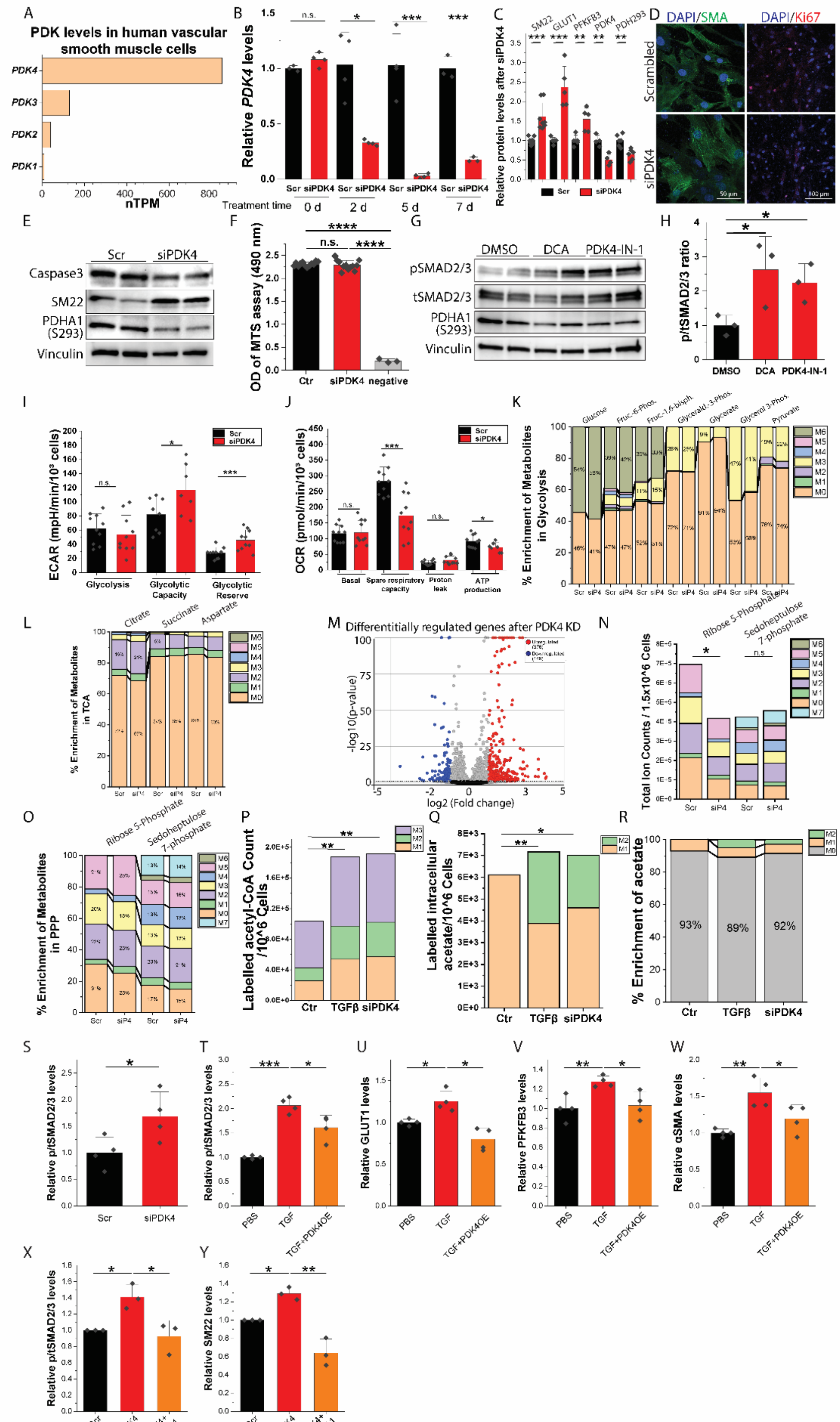

A
PDK levels in human vascular smooth muscle cells
PDK4
PDK3
PDK2
PDK1
nTPM
B
Relative PDK4 levels
n.s.
*
***
***
Scr siPDK4 Scr siPDK4 Scr siPDK4 Scr siPDK4
Treatment time 0 d 2 d 5 d 7 d
C
SM22 GLUT1 PFKFB3 PDK4 PDH293
Relative protein levels after siPDK4
Scr siPDK4
D
DAPI/SMA
DAPI/Ki67
Scrambled
siPDK4
E
Scr siPDK4
Caspase3
SM22
PDHA1 (S293)
Vinculin
F
****
n.s.
****
OD of MTS assay (490 nm)
Ctr siPDK4 negative
G
DMSO DCA PDK4-IN-1
pSMAD2/3
tSMAD2/3
PDHA1 (S293)
Vinculin
H
p/tSMAD2/3 ratio
DMSO DCA PDK4-IN-1
I
ECAR (mpH/min/10³ cells)
Scr
siPDK4
n.s.
*
***
Glycolysis
Glycolytic Capacity
Glycolytic Reserve
J
OCR (pmol/min/10³ cells)
Scr
siPDK4
n.s.
***
n.s.
*
Basal
Spare respiratory capacity
Proton leak
ATP production
K
Glucose
Fruc.-6-Phos.
Fruc.-1,6-bisph.
Glyceraldehyde-3-Phos.
Glycerate
Glycerol 3-Phos.
Pyruvate
% Enrichment of Metabolites in Glycolysis
M6 M5 M4 M3 M2 M1 M0
Scr siP4 Scr siP4 Scr siP4 Scr siP4 Scr siP4 Scr siP4 Scr siP4
L
Citrate
Succinate
Aspartate
% Enrichment of Metabolites in TCA
M6 M5 M4 M3 M2 M1 M0
Scr siP4 Scr siP4 Scr siP4
M
Differentitially regulated genes after PDK4 KD
-log10(p-value)
log2 (Fold change)
N
Ribose 5-Phosphate
Sedoheptulose 7-phosphate
*
n.s
Total Ion Counts / 1.5x10^6 Cells
M6 M5 M4 M3 M2 M1 M0 M7
Scr siP4 Scr siP4
O
Ribose 5-Phosphate
Sedoheptulose 7-phosphate
% Enrichment of Metabolites in PPP
M6 M5 M4 M3 M2 M1 M0 M7
Scr siP4 Scr siP4
P
**
**
Labelled acetyl-CoA Count /10^6 Cells
M3 M2 M1
Ctr TGFβ siPDK4
Q
**
*
Labelled intracellular acetate/10^6 Cells
M2 M1
Ctr TGFβ siPDK4
R
% Enrichment of acetate
M2 M1 M0
93% 89% 92%
Ctr TGFβ siPDK4
S
*
Relative p/tSMAD2/3 levels
Scr siPDK4
T
***
*
Relative p/tSMAD2/3 levels
PBS TGF TGF+PDK4OE
U
*
*
Relative GLUT1 levels
PBS TGF TGF+PDK4OE
V
**
*
Relative PFKFB3 levels
PBS TGF TGF+PDK4OE
W
**
*
Relative αSMA levels
PBS TGF TGF+PDK4OE
X
*
*
Relative p/tSMAD2/3 levels
Scr siPDK4 siPDK4+ siSLC2A1
Y
*
**
Relative SM22 levels
Scr siPDK4 siPDK4+ siSLC2A1

Supplementary figure 2 Metabolic effects of PDK4 knockdown in HASMCs

(**A**) Transcripts per million (nTPM) of four PDK isoforms in human vascular aorta.

(**B**) Time course qPCR analyses of relative PDK4 levels upon siPDK4 treatment for 0, 2, 5 and 7 days. Each dot represents one technical replicate from a representative experiment with quadruplicate.

(**C**) Quantification of SM22, GLUT1, PFKFB3, PDK4 and PDH293 after PDK4 KD for 7 days. Normalized and pooled results from 3-5 independent repeats. Each dot represents one technical replicates.

(**D**) Immunostaining of αSMA and Ki67 of HASMCs after PDK4 KD for 7 days.

(**E-F**) Western blot of Caspase-3 and MTS assay in HASMC after PDK4 KD for 7 days. Normalized and pooled results from 3 independent repeats. Each dot represents one technical replicate.

(**G-H**) Representative Western blot and quantification of SMAD2/3 phosphorylation after DCA or PDK4-IN-1 treatment for 3 days. Each dot represents one technical replicate.

(**I-J**) Seahorse analyses of (I) glycolysis, glycolytic capacity and glycolytic reserve and (J) basal oxygen consumption, spare respiratory capacity, proton leak and ATP production of HASMCs after PDK4 KD for 7 days. Representative results were displayed with 8 technical replicates. Each dot represents one technical replicate.

(**K-L**) LC-MS metabolomics analysis of key metabolites in HASMCs treated with C^{13}-glucose (U-13C6-glucose, 10 mM) for 24 h after PDK4 KD for 7 days. M# represents the number of C^{13}-labeled carbon in each metabolite. Note that the % of C^{13} labelled metabolites in glycolysis (K) were increased by TGFβ, whereas that of key metabolites in the TCA cycle (L) were not increased. Representative results were displayed with quadruplicates.

(**M**) Volcano plot from bulk RNA-seq analysis of HASMC contractile phenotype gene expression under 20 nM siPDK4 treatment for 7 days (n=3).

(**N-O**) Total ion counts (N) and percentage (O) of C^{13}-labeled metabolites in PPP. HASMCs treated with C^{13}-glucose (U-13C6-glucose, 10 mM) for 24 h after PDK4 KD for 7 days. Representative results were displayed with quadruplicates.

(**P**) Total ion counts of C^{13}-glucose derived-Ac-CoA quantification by LC-MS in HASMCs treated with TGFβ or PDK4 knockdown for 7 days. M# refers to the C^{13} number in one molecule. Representative results were displayed with quadruplicates.

(**Q-R**) Total ion counts and % of C^{13} labelled intracellular acetate in HASMCs treated with TGFβ or PDK4 KD for 7 days. (Q) includes the non-labelled acetate (M0). Note that no M2 labelled acetate was observed in control group. One sample t-test was performed for M2 acetate in the TGFβ or PDK4 KD group. Representative results were displayed with quadruplicates.

(**S**) Quantification of phosphorylated to total level of SMAD2/3 after PDK4 KD for 7 days. Normalized and pooled results from 3 independent repeats. Each dot represents one technical replicate.

(**T-W**) Quantification of phosphorylated to total level of SMAD2/3, GLUT1, PFKFB3, αSMA levels after 3-day treatment with 10ng/mL TGFβ with PDK4 adenovirus or control virus for 3 days. Normalized and pooled results from 3 independent repeats. Each dot represents one technical replicate.

(**X-Y**) Quantification phosphorylated to total level of SMAD2/3 and SM22 levels after 7 days of PDK4 KD or PDK4 and GLUT1 double KD. Normalized and pooled results from 3 independent repeats. Each dot represents one repeat.

One-way ANOVA was used for statistical analyses of (B, D, F, H, I, J, N, P, Q, S, T, U, V and W). Paired-sample t-test was used in (X and Y).

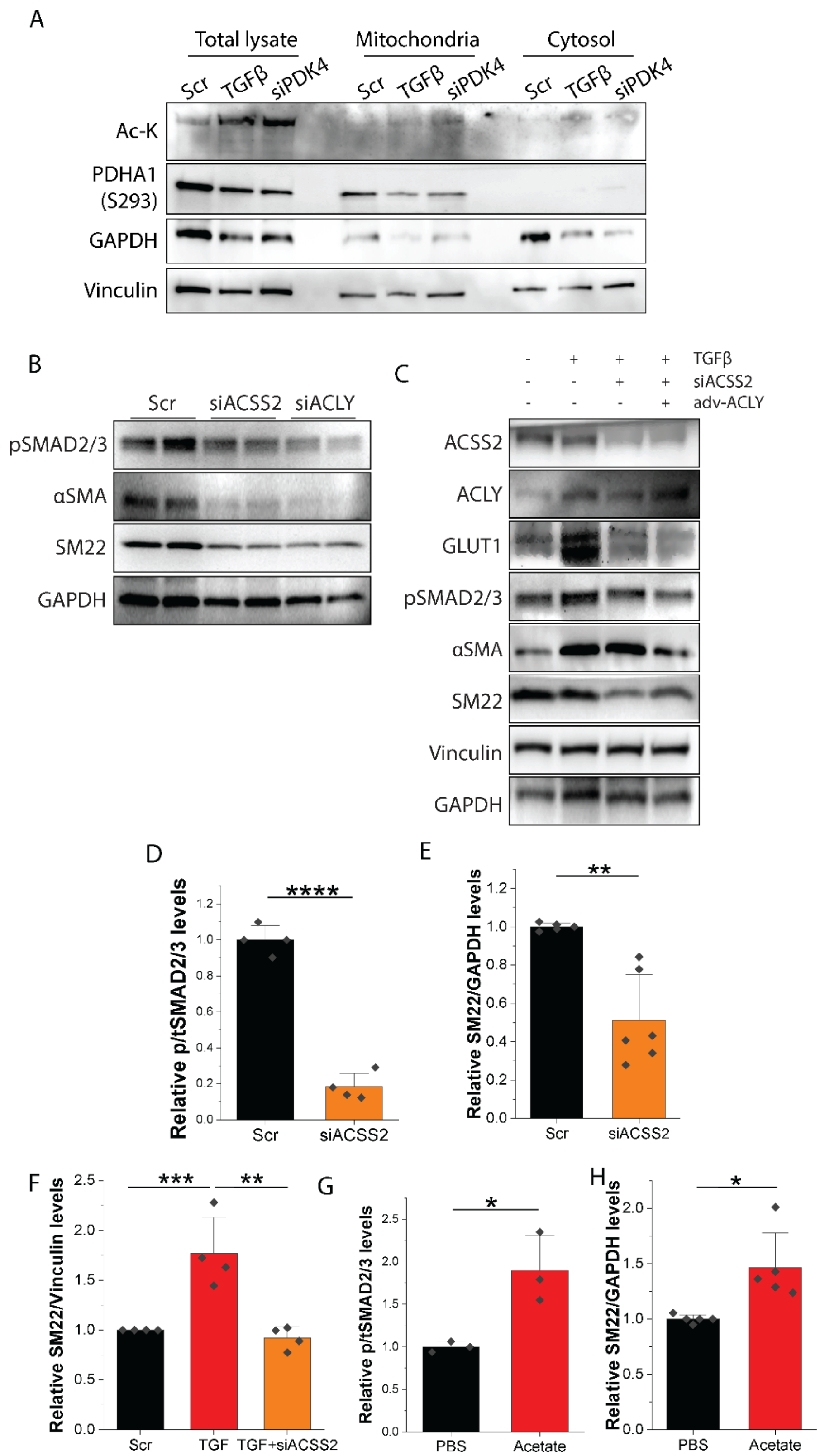

A
Total lysate
Mitochondria
Cytosol
Scr TGFβ siPDK4
Scr TGFβ siPDK4
Scr TGFβ siPDK4
Ac-K
PDHA1 (S293)
GAPDH
Vinculin
B
Scr
siACSS2
siACLY
pSMAD2/3
αSMA
SM22
GAPDH
C
TGFβ
siACSS2
adv-ACLY
ACSS2
ACLY
GLUT1
pSMAD2/3
αSMA
SM22
Vinculin
GAPDH
D
Relative p/tSMAD2/3 levels
****
Scr
siACSS2
E
Relative SM22/GAPDH levels
**
Scr
siACSS2
F
Relative SM22/Vinculin levels
***
**
Scr
TGF
TGF+siACSS2
G
Relative p/tSMAD2/3 levels
*
PBS
Acetate
H
Relative SM22/GAPDH levels
*
PBS
Acetate

Supplementary figure 3 Analysis of Subcellular distribution of protein acetylation

(**A**) Western blotting of acetylated protein, using anti-acetylated lysine (Ac-K). Phosphorylated PDHA1 (S293) is used as mitochondria marker. GAPDH is mainly in the cytoplasm.

(**B**) Western blotting of pSMAD2/3 and SMC contractile markers (αSMA and SM22) upon ACSS2 or ACLY knockdown for 3 days.

(**C**) Rescuing experiments of adenoviral overexpressing ACLY (adv-ACLY) on TGFβ signaling (pSMAD2/3), SMC contractile phenotype (αSMA and SM22) and glucose uptake (GLUT1), after ACSS2 knockdown.

(**D-H**) Quantification of phosphorylated to total ratio of SMAD2/3 and SM22 levels, normalized to GAPDH or Vinculin, to investigate the effects of ACSS2 KD and 10 mM acetate treatment on HASMC contractile phenotype. One-way ANOVA was used for statistical analyses for (D, E, G and H, each dot represents one technical replicate, normalized and pooled from 3 independent repeats). Paired-sample t-test were applied to (F, each dot represents one biological replicate from 4 independent repeats).

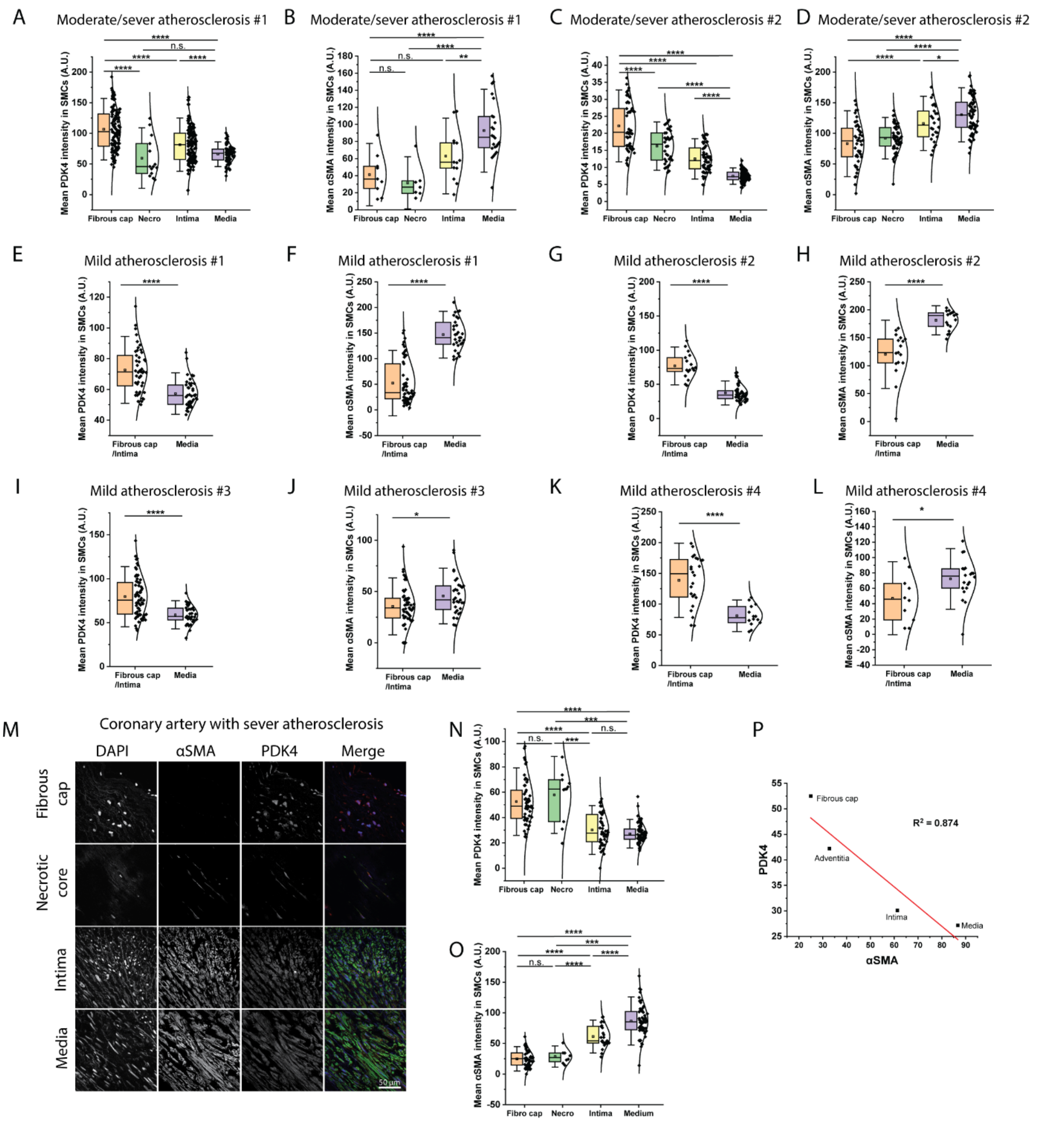

Supplementary figure 4 Upregulated PDK4 in atherosclerotic SMCs in human aortae and coronary arteries.

(**A-L**) Quantifications of PDK4 and αSMA staining in human ascending aorta from human patients either with moderate/sever (A-D) or mild (E-L) atherosclerosis. Each dot represents one SMC.

(**M-O**) Representative images (M), quantification (N-O) of PDK4 and αSMA intensity of coronary artery from a patient with sever atherosclerosis. Each dot represents one SMC in indicated regions.

(**P**) Linear fitting comparing mean PDK4 and αSMA intensity from different locations in coronary artery. Each dot represents one region indicated.

One-way ANOVA was used for statistical analyses

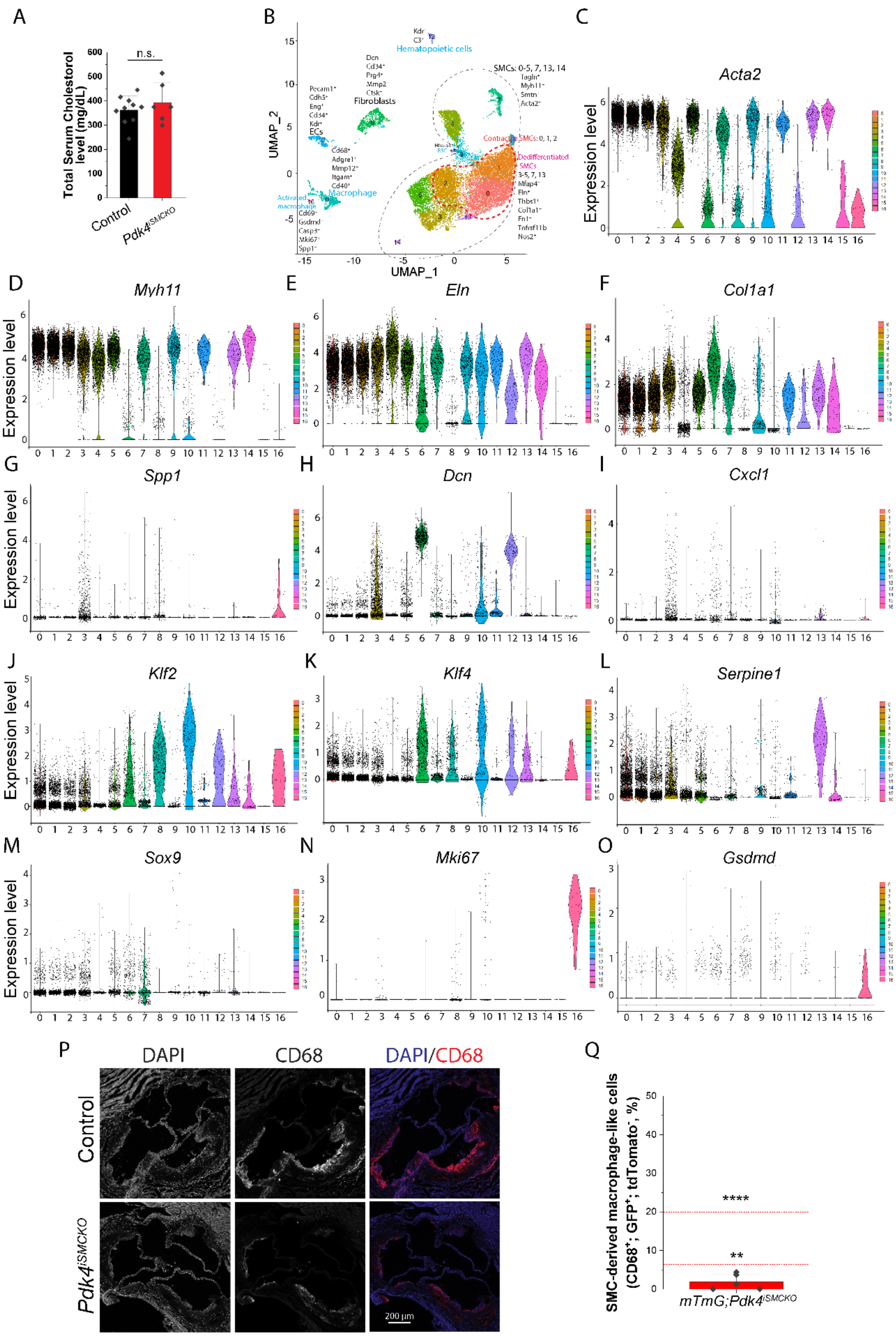

Supplementary figure 5 *Pdk4* knockout ameliorates atherosclerosis and maintains SMC

contractile phenotype in *ApoE*$^{-/-}$ mice.

(**A**) Comparison of total serum cholesterol levels between control and Pdk4iSMCKO mice after 4-month HFD feeding. Each dot represents one mouse.

(**B**) FeaturePlot analyses of aortic cells from both control and *Pdk4*^{iSMCKO} mice, with enriched mRNAs in each cluster.

(**C-O**) Violin plots showing expressions of key cell differentiation markers among all the clusters. Each dot represents one cell in each cluster.

(**P**) Representative images of CD68 staining of aortic root cross-sections from control and *Pdk4*^{iSMCKO} mice.

(**Q**) Quantification of SMC-derived fraction of CD68^{+} macrophages from five mTmG;*Pdk4*^{iSMCKO} mice, with green fluorescence expressing (GFP^{+}) and without TdTomato. One sample t-test was performed comparing 5 mice with the reported mean 20% SMC-derived macrophages by Owsiany *et al*. (*32*), or 6.3% as reported by Chen *et al.* (*9*). Each dot represents one mouse.

Supplementary Table

Supplementary Table 1: Gene expression profile of clusters from scRNAseq

Cluster#	0	1	2	3	4	5	6	7	8	9	10	11	12	13	14	15	16
SMC/Contractile	+++	+++	+++	+++	++	+++	-	++	-	++	-	++	-	++	++	-	-
SMC/Synthetic	+	+	+	+++	+	++	+++	++	-	+	-	++	-	++	+	-	-
Macrophages	-	-	-	-	-	-	-	-	+++	-	-	-	-	-	-	-	+++
Innate Immune	-	-	-	++	-	-	++	-	+	-	-	-	-	+	-	-	+
Fibroblasts	-	-	-	+	-	-	+++	-	-	-	+	+	++	-	-	-	-
Endothelial cells	-	-	-	++	-	-	+	+	-	-	+++	-	-	+	-	-	-
Osteoblast-like	-	-	-	++	-	-	+	+	+	-	-	-	-	-	-	-	+
Stem cell like	-	-	-	+	-	-	++	-	+	-	+	-	+++	-	-	-	+

+++ strongest expression, ++ medium, + low, - No expression

Contractile SMC clusters: 0, 1 and 2

Synthetic SMC clusters: 3, 4, 5, 7, 9, 11, 13 and 14

Macrophage clusters: 8 and 16

Fibroblasts: 6

Endothelial cells: 10

Hematopoietic cells: 12

Red blood cells: 15

Supplementary Table 2: Sequences (5'-3') of primers used in qPCR

Gene	Forward primers	Reverse primers
ACTA2	CTATGCCTCTGGACGCACAACT	CAGATCCAGACGCATGATGGCA
CALD	CTGTTCCTGCTGAAGGTGTACG	CCTACCTTCAAGCCAGCAGTTTC
CNN1	CCAACGACCTGTTTGAGAACACC	ATTTCCGCTCCTGCTTCTCTGC
HK1	CTGCTGGTGAAAATCCGTAGTGG	GTCCAAGAAGTCAGAGATGCAGG
LDHB	CCTCAGATCGTCAAGTACAGTCC	ATCACGCGGTGTTTGGGTAAT
MYL9	GGATGTGATTCGCAACGCCTTTG	CGGTACATCTCGTCCACTTCCT
PDK4	GGAGCATTTCTCGCGCTACA	ACAGGCAATTCTTGTCGCAAA
PKM	ATGTCGAAGCCCCATAGTGAA	TGGGTGGTGAATCAATGTCCA
SLC2A1	ATTGGCTCCGGTATCGTCAAC	GCTCAGATAGGACATCCAGGGTA
TAGLN	TCCAGGTCTGGCTGAAGAATGG	CTGCTCCATCTGCTTGAAGACC
β-ACTIN	ATCAAGATCATTGCTCCTCCTGAG	CTGCTTGCTGATCCACATCTG
